# Disparity in the use of Alzheimer's disease treatment in Southern Brazil

**DOI:** 10.1038/s41598-023-36604-4

**Published:** 2023-06-12

**Authors:** Maisa De Marco, Ana Laura Brandi, Andrei Bieger, Bárbara Krug, Analuiza Camozzato, Paulo D. Picon, Marcia Lorena Fagundes Chaves, Raphael Machado Castilhos

**Affiliations:** 1 Graduate Program in Medical Sciences, Universidade Federal do Rio Grande Sul, Porto Alegre, Rio Grande do Sul 90035003 Brazil; 2 Faculdade de Medicina, Universidade Federal do Rio Grande Sul, Porto Alegre, Rio Grande do Sul 90035003 Brazil; 3 Biochemistry Department, Universidade Federal do Rio Grande Sul, Porto Alegre, Rio Grande do Sul 90040060 Brazil; 4 Secretaria Estadual de Saúde do Estado do Rio Grande do Sul, Porto Alegre, Rio Grande do Sul 90110150 Brazil; 5 Department of Psychiatry, Universidade Federal de Ciências da Saúde de Porto Alegre (UFCSPA), Porto Alegre, Rio Grande do Sul 90050170 Brazil; 6 Department of Internal Medicine, Universidade Federal do Rio Grande do Sul (UFRGS), Porto Alegre, Rio Grande do Sul 90035903 Brazil; 7 Cognitive and Behavioral Neurology Center, Division of Neurology, Hospital de Clínicas de Porto Alegre, Ramiro Barcelos Street 2350, Porto Alegre, Rio Grande do Sul 90035903 Brazil

**Keywords:** Alzheimer's disease, Dementia

## Abstract

Alzheimer's disease (AD) treatment is freely available in the Brazilian public health system. However, the prescription pattern and its associated factors have been poorly studied in our country. We reviewed all granted requests for AD treatment in the public health system in October 2021 in the Rio Grande do Sul (RS) state, Southern Brazil. We performed a spatial autocorrelation analysis with the population-adjusted patients receiving any AD medication as the outcome and correlated it with several socioeconomic variables. 2382 patients with AD were being treated during the period analyzed. The distribution of the outcome variable was not random (Moran's I 0.17562, *P* <.0001), with the most developed regions having a higher number of patients/100,000 receiving any AD medication. We show that although AD medications are available through the public health system, there is a clear disparity between regions of RS state. Factors related to socioeconomic development partly explain this finding.

## Introduction

Dementia is a leading cause of disability in the elderly, currently affecting around 60 million people worldwide, with an estimated prevalence to triple by 2050^1^. Alzheimer's disease (AD) is the main cause of dementia, accounting for 60–70% of cases, and is characterized clinically by progressive decline in cognition, behavior and functionality and biologically by brain accumulation of abnormal amyloid β and hyperphosphorylated tau^2^.

The pharmacological treatment for AD is fundamentally symptomatic and consists mainly of three acetylcholinesterase inhibitors (AChEi) (donepezil, rivastigmine and galantamine) and one antagonist of *N*-methyl-*D*-aspartate (NMDA) receptor, memantine. The effect size of these drugs is generally modest, although some patients may experience a more robust benefit^3^. In Brazil, these medications have been approved since 2002 and are freely available in the public health system (Sistema Único de Saúde, SUS) through an evaluation conducted by a specific program known Specialized Component of Pharmaceutical Assistance). Under this program, all requests must meet specific criteria based on Clinical Practice Guidelines^4^. In the last version of that Guideline, AChEi are recommended to mild and moderate disease and memantine to moderate and severe stages^5,6^.

Although these medications are available throughout the country, there may be barriers to access of these treatments. Bureaucratic intricacies, geographic accessibility and other socioeconomic factors can affect the provision of these medications^6–8^. Patient-related factors such as formal education and race also appear to contribute to limited access^9,10^. In addition, the underdiagnosis of dementia in Brazil is probably a central component for the medications access, a phenomenon related, among other causes, to scarcity of physicians and their poor capacity to perform these diagnoses^11^. In a previous Brazilian study, Moraes et al. showed that there were more prescriptions for AD medications in the more developed regions of the country^12^, a probable indicator of the fragility of the diagnostic and therapeutic process of patients with dementia.

Although Brazil is a continental country with a well-structured and comprehensive public health system, few studies have evaluated possible disparities in the AD medications prescriptions and none were performed in Rio Grande do Sul (RS), the 6th most populous in the country. Therefore, our aim is to evaluate the spatial pattern of pharmacological treatment for AD in RS and possible factors associated with this pattern.

## Methods

We performed a study with two different designs. A cross-sectional study, in which we analyzed the profile of patients receiving medication for AD in October 2021 in the state of RS, Brazil. And an ecological study, where we analyzed the prescription profile of AD medications in all municipalities of RS during this period. We reviewed all requests granted for medications for AD in the Health Department of the State of RS in October 2021. We evaluated requests for the four medications available in the public health system, in any formulation or dose: donepezil, rivastigmine, galantamine and memantine. As recommended by the National Guidelines, in the state of RS the requests for medication for AD through the public system are initially performed by the attending physician through a web-based system called AME (Administração de Medicamentos Especiais), managed by the RS State Health Secretariat. Requests from all over the state are evaluated through an administrative process and centrally approved or denied by a trained team located in the Hospital de Clínicas de Porto Alegre, Porto Alegre, the state capital. The established guidelines and process are necessary to prevent the irrational use of these drugs^6^. Within this administrative process, minimum clinical data, such as Mini Mental State Examination (MMSE)^13,14^ and Clinical Dementia Rating (CDR) scale^15,16^ should be added, in addition to laboratory and imaging tests that attest that the patient has AD as the probable etiology for the dementia syndrome.

### Study variables

In the cross-sectional study we evaluated the following variables: patients' age, sex, educational attainment, city of origin, total score on MMSE and CDR scales and type/dose of AD medication prescribed. All these variables were extracted from the web-based system (AME), the administrative system through each request is made and judged.

In the ecological analysis we evaluated the total number of patients/100,000 inhabitants (outcome variable) for each municipality who were receiving any AD medication. We performed a spatial autocorrelation analysis to assess the distribution pattern of the variable patients/100,000/municipality in the state of RS in October 2021. After this evaluation, we correlated the outcome variable with the following factors of each municipality: percentage of female patient sex, mean patients’ age, mean patients’ MMSE, percentage of patients ≥ 65 years, death records by AD, human development index (HDI), gross domestic product (GDP) per capita, income per capita, proportion of rural households, percentage of patients with elementary, high school, university education and illiterates, total number of physicians and the median time (in years) as a physician, a measure of experience as a physician. All variables related to the municipalities were obtained from the Department of Informatics of the Unified Health System (DATASUS) of Brazil^17^ and the Atlas of Human Development of Brazil^18^, both freely available. The experience as a physician was extracted from the open access State Medicine Council database^19^.

### Statistical analysis

Categorical variables were described by frequency and continuous ones by mean (standard deviation, SD) or median (interquartile range, IQR), according to their distribution. The association between the continuous variables was evaluated by the Spearman coefficient. The pattern of distribution among the RS municipalities was evaluated by spatial autocorrelation using Moran's I index. All analyses were performed using the built-in functions and the packages "sf", "tmap" and "ggplot2" of the R software (V 4.2.1).

### Ethics approval

This study followed the Declaration of Helsinki and was approved by the Research Ethics Committees of the Hospital de Clínicas de Porto Alegre (CAAE 43246921.7.0000.5327) and the State Health Secretariat (CAAE 43246921.7.3001.5312).

### Consent to participate

A consent was not necessary as the patient’s data were obtained from databases, whose access was granted by the Health Department of the Rio Grande do Sul State.

## Results

In October 2021, in the state of RS, Brazil, 2,382 AD patients were using any of the four drugs through the Brazilian public health system. The majority, 65.6% (1,562), were female, had a median age of 79 (73–84) years and 71.7% (1036) had incomplete elementary education. The patients' MMSE median was 15 (12–18) and most had a CDR = 2 (55.2%, 1133) (Table 1). The most prescribed medication was donepezil, 42.9% (1014), alone or in combination with memantine. We described all AD medications used in the sample in Online Resource 1. Most professionals who prescribed these medications were experienced physicians, with a median professional activity of 18 (9–30) years.

**Table 1:** Sociodemographic and clinical characteristics of patients receiving medication for Alzheimer's disease in the state of Rio Grande do Sul.

Variable	
Total sample	2,382
Female (%)	1,562 (65.5)
Age median (IQR)	79 (73–84)
Education (%)	
Illiteracy	96 (6.6)
Elementary school complete	134 (9.3)
Elementary school incomplete	1,036 (71.7)
High school complete	57 (3.9)
High school incomplete	51 (3.5)
University education complete	49 (3.4)
University education incomplete	22 (1.5)
MMSE	
median (IQR)	15 (12–18)
Clinical dementia rating (CDR) Scale (%)	
CDR 0	3 (0.1)
CDR 0.5	37 (1.8)
CDR 1	830 (40.4)
CDR 2	1,133 (55.2)
CDR 3	51 (2.5)

*MMSE* Mini mental state examination.

**Figure 1:**
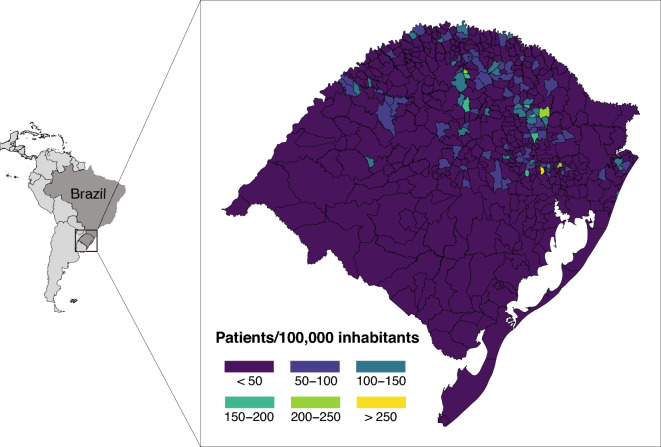
Choropleth map of spatial distribution of patients/100,000 inhabitants receiving Alzheimer's disease medications in the state of Rio Grande do Sul, Brazil. Maps created with R software, version 4.2.2, and Adobe Illustrator, version 27.5.

**Figure 2:**
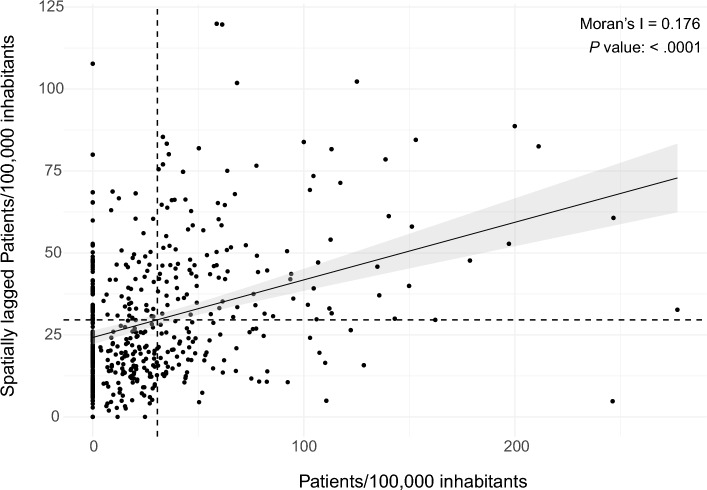
Moran scatter plot, showing the correlation between the patients/100,000 variable and its spatial lag.

**Figure 3:**
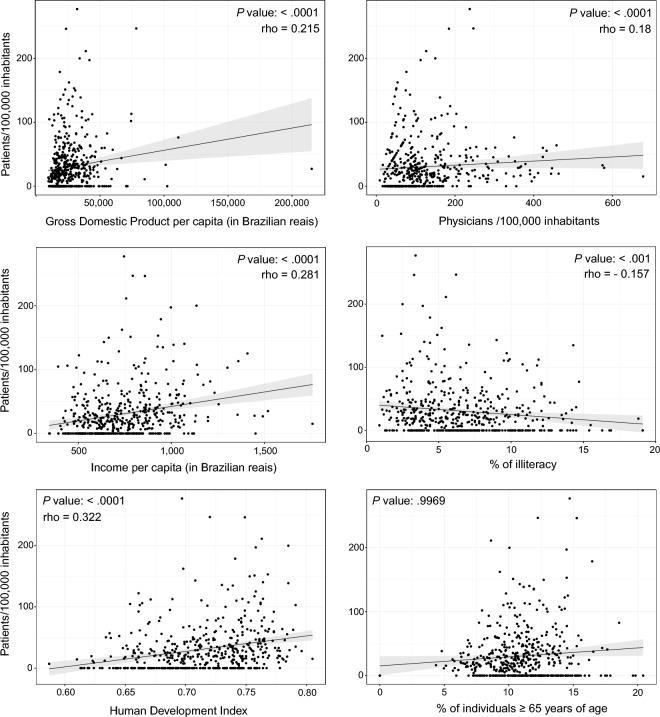
Scatter plots between patients/100,000 inhabitants and socioeconomic variables.

Most municipalities had at least one patient receiving an AD medication (339/498, 68.2%). The median of patients/100,000/municipality using any AD medication was 19.4 [0–42.8], ranging from 0 to 277. The sociodemographic of RS state municipalities are shown in Table 2. We found that the variable patient/100,000/municipality was clustered, with the Moran's I of 0.17562 (*P* < .001) (Figs. 1 and 2), with the North and Northeast regions concentrating the prescriptions. As the distribution of this variable was not random, we correlated it with municipalities sociodemographic variables. Several variables had weak correlation with patients/100,000/municipality such as HDI (rho = 0.32), GDP per capita (rho = 0.21), income per capita (rho = 0.281), physicians/100,000 inhabitants (rho = 0.17) and % of illiteracy (rho =−0.16) (Fig. 3). Table 3 presents the correlation of all variables.

**Table 2:** Sociodemographic- and patient-related variables from municipalities in the state of Rio Grande do Sul.

	Median (IQR)	Range
Patients/100,000 inhabitants	19.4 (0–42.8)	0–277
HDI	0.717 (0.685–0.746)	0.587–0.805
GDP per capita (in Brazilian reais)	22,475 (17,509–31,372)	9,597–215,394
Income per capita (in Brazilian reais)	714.3 (595.9–862.7)	343.1–1758.3
% Rural domicile	47 (21–66.8)	0–98.6
Education		
% Illiteracy	6.1 (3.9–8.5)	0.9–19.1
% Elementary	35.4 (28.4–43.3)	14.1–73.5
% High school	20.5 (15.8–26.4)	5.3–57.8
% University	5.4 (3.9–7.5)	0.4–25.9
% Female	66.7 (50–100)	0–100
Patients' age	78 (75–81)	57–95
% age ≥ 65 years	10.9 (9.4–12.5)	0–20.4
MMSE	15 (13–17)	2–30
Deaths from AD/100,000 inhabitants	24.1 (15.7–38.6)	2.6–261.8
Physicians/100,000 inhabitants	97.46 (60.8–156.2)	9–678
Experience as a physician (in years)	18.7 (11.8–25.2)	0–53

*AD* Alzheimer's disease, *GDP* Gross domestic product, *HDI* Human development index, *MMSE* Mini mental state examination.

**Table 3:** Correlation between patients/100,000/municipality and sociodemographic-, patient- and medical-related variables.

	Spearman’s rho (*P* value) *
HDI	0.32 (< .0001)
GDP per capita	0.21 (< 0.001)
Income per capita	0.28 (< 0.0001)
% Rural domicile	ns
Education	
% Illiteracy	−0.16 (< 0.001)
% Elementary	0.19 (< 0.0001)
% High school	0.19 (< 0.0001)
% University	0.17 (< 0.001)
% Female	ns
Patients' age	ns
% age ≥ 65 years	ns
MMSE	ns
Deaths from AD/100,000 inhabitants	ns
Physicians/100,000 inhabitants	0.17 (< 0.001)
Experience as a physician (in years)	ns

^*^P value threshold after Bonferroni correction (< .002), *ns* not significant, *AD* Alzheimer's disease, *GDP* Gross domestic product, *HDI* Human development index, *MMSE* Mini mental state examination.

## Discussion

In our study, we were able to assess the pattern of AD medications granted by the Brazilian health system in the state of RS. We showed that although these medications are available to all patients through the Brazilian public health system, there is a clear disparity between regions. Previous Brazilian studies had already shown that there was some disparity in the supply of these medication, but none of them had evaluated a whole state or attempted to assess factors related to possible disparities^7,12^; moreover, the evaluation through spatial autocorrelation is innovative in this regard, since it is the first time that an ecological approach had been performed to evaluate the spatial distribution of drugs for AD.

We showed that the number of patients/100,000 inhabitants did not have a random pattern (Moran's I of 0.17562, *P* < .001), with the north and northeast regions having more patients/prescriptions, adjusted for the population, than other regions of the state. Such an uneven pattern of prescription of AD drugs has already been demonstrated in other countries. Hausner et al. evaluated the distribution of dementia drugs in several European countries and showed that southern countries had fewer patients using these drugs^9^. As the pattern was not random, we were able to correlate the variable patients/100,000 inhabitants with some sociodemographic characteristics of the municipalities. Several socioeconomic development variables showed weak correlation with the outcome variable, such as the Human Development Index and Gross Domestic Product. This finding is similar to previous studies that showed that economic disadvantages were determinant for the distribution of AD medications. In an Australian study, patients from more developed regions were 2.4 times more likely to receive some medication for AD than less developed regions^8^. Copper et al., despite not having assessed a regional pattern of prescription in London, showed that people who did not own a home receive less medication for AD from the British public health system^20^.

Another factor related to the number of patients/100,000 inhabitants was the formal education. We showed that there was a positive correlation between the percentage of patients who had elementary, high school and university education with the outcome variable. This finding corroborates previous studies that showed that years of formal education were related to the prescription of AD medications. In a large Swedish study, Johnell et al. identified that patients with more that 15 years of schooling receive more prescriptions than those with less than 9 years of education^21^. Similar findings were seen in US^22^ and UK^23^ patients. Certainly, years of formal education is a marker of sociodemographic development and therefore it is not surprising that these variables are also related to the distribution of AD medications.

Remoteness and living in rural areas are factors usually associated with probability of prescription of AD medications, either with fewer prescriptions^24^ or more prescriptions^25^. In our study, there was no association between the percentage of rural households and the number of patients/100,000 inhabitants. A possible explanation for this finding is that the supply of AD medications is carried out at the health department of each municipality and not at a regional center, i.e., access is close and similar in every town.

Finally, we tried to assess whether factors related to the patients or to the physicians who prescribed the medications could correlate with the number of patients receiving AD medications in each municipality. Patient's age, sex, MMSE scores and AD deaths records were not associated with the outcome. These are in line with previous findings^23,26^. One hypothesis is that the characteristics of the patients are usually similar between different regions, therefore not being a determining factor for the spatial distribution pattern of AD medications. We showed that the total number of physicians/100,000 per municipality, but not the experience as a physician, was weakly associated (rho=0.17) with the number of patients receiving any medication. Although we did not find a previous study that showed a similar result, we consider it to be a straightforward finding, since fewer physicians in a municipality indicate a lower chance of these drugs being prescribed.

One might argue that all variables that were associated with the number of patients/100,000 inhabitants had weak correlation coefficients. However, this finding may be related to the fact that there are multiple determining factors in the chain of events necessary for a patient with AD to receive one of these medications. The correct clinical suspicion by family members, the availability and access to health services capable of performing the diagnosis and the correct completion of the form by the physician, all these steps can be barriers to accessing these medications. We hypothesize that all these steps may work more quickly and efficiently in more developed regions.

The sociodemographic and clinical characteristics of patients receiving an AD drug in our study were similar to what had been seen in other studies, as most were women with low education^27,28^. In addition, most of the patients had moderate dementia (CDR = 2). Although we know that patients with dementia usually have a diagnostic delay in Brazil^29^, we could not assess whether this delay was associated with our results. We also found 3 patients with CDR = 0 receiving AD medication. We were unable to assess whether it was a typo in the web system or an error in evaluating the request. Regarding the type of medication prescribed, most patients were using donepezil, a change in prescription pattern in the state of RS, since a previous study performed in 2010 showed that rivastigmine was the most prescribed medication, 86.1%^6^. However, it is similar to a more recent Brazilian study^12^.

Our study has some limitations. First and foremost, we have only evaluated AD medications through the public health system, and they can be purchased at pharmacies with a prescription. Thus, it is possible that the prescription pattern can be different in the state of RS. However, if we consider the economic difficulties of our country, we expected that, in relation to the treatment of AD, the less developed regions of the state would in fact be the ones that depended most on public health. We found the opposite, suggesting that the process of diagnosis and treatment of less developed regions was more determinant of the pattern we found. In addition, as we obtained the data from records of deferred processes filled in by prescribing physicians, it is possible that some information was recorded incorrectly. Finally, we were unable to assess the race/ethnicity of the patients, as this information was not available in the database. This assessment could have added additional perspective on the disparity.

In conclusion, we showed that the pattern of prescription of AD medications by the public health system in the state of RS, Brazil is uneven and more socioeconomically developed regions have higher frequency of prescriptions. Assessing the roots of this disparity can lead to public policy changes aiming to improve diagnosis and treatment of patients with Alzheimer's disease here and abroad.

### Supplementary Information

Supplementary Information.

## Data Availability

The datasets generated during and/or analyzed during the current study are available from the corresponding author on reasonable request.
