# Author Correction: Disparity in the use of Alzheimer's disease treatment in Southern Brazil

**DOI:** 10.1038/s41598-023-41252-9

**Published:** 2023-08-29

**Authors:** Maisa De Marco, Ana Laura Brandi, Andrei Bieger, Bárbara Krug, Analuiza Camozzato, Paulo D. Picon, Marcia Lorena Fagundes Chaves, Raphael Machado Castilhos

**Affiliations:** 1 Graduate Program in Medical Sciences, Universidade Federal do Rio Grande Sul, Porto Alegre, Rio Grande do Sul 90035003 Brazil; 2 Faculdade de Medicina, Universidade Federal do Rio Grande Sul, Porto Alegre, Rio Grande do Sul 90035003 Brazil; 3 Biochemistry Department, Universidade Federal do Rio Grande Sul, Porto Alegre, Rio Grande do Sul 90040060 Brazil; 4 Secretaria Estadual de Saúde do Estado do Rio Grande do Sul, Porto Alegre, Rio Grande do Sul 90110150 Brazil; 5 Department of Psychiatry, Universidade Federal de Ciências da Saúde de Porto Alegre (UFCSPA), Porto Alegre, Rio Grande do Sul 90050170 Brazil; 6 Department of Internal Medicine, Universidade Federal do Rio Grande do Sul (UFRGS), Porto Alegre, Rio Grande do Sul 90035903 Brazil; 7 Cognitive and Behavioral Neurology Center, Division of Neurology, Hospital de Clínicas de Porto Alegre, Ramiro Barcelos Street 2350, Porto Alegre, Rio Grande do Sul 90035903 Brazil

Correction to: *Scientific Reports* https://doi.org/10.1038/s41598-023-36604-4, published online 12 June 2023

Andrei Bieger was omitted from the author list in the original version of this Article.

The Author Contributions section now reads:

“All authors contributed to the study conception and design. Material preparation, data collection and analysis were performed by [M.M.], [A.L.B.] and [R.M.C]. R codes were developed and implemented by [A.B.]. The first draft of the manuscript was written by [M.M.] and [R.M.C]; and all authors commented on previous versions of the manuscript. All authors read and approved the final manuscript.”

The original Article and accompanying Supplementary Information file have been corrected.

