# Supplementary material for: Disparity in the use of Alzheimer's disease treatment in Southern Brazil
Source: Sci Rep. 2023 Jun 12;13:9555. doi: 10.1038/s41598-023-36604-4 (PMC10261112; doi:10.1038/s41598-023-36604-4)
Supplement: Supplementary file 1 — Supplementary Information. [file 41598_2023_36604_MOESM1_ESM.pdf]

Scientific Reports

Disparity in the use of Alzheimer's disease treatment in Southern Brazil.

Maisa De Marco [a], Ana Laura Brandi [b], Andrei Bieger [c,d], Bárbara Krug [e], Analuiza Camozzato [f], Paulo D. Picon [g], Marcia Lorena Fagundes Chaves [g,h], Raphael Machado Castilhos [a,h]

[a] Graduate Program in Medical Sciences, Universidade Federal do Rio Grande Sul, Porto Alegre, Rio Grande do Sul, Brazil; Postal Code: 90035003;

[b] Faculdade de Medicina, Universidade Federal do Rio Grande Sul, Porto Alegre, Rio Grande do Sul, Brazil; Postal Code: 90035003;

[c] Biochemistry Department, Universidade Federal do Rio Grande Sul, Porto Alegre, Rio Grande do Sul, Brazil; Postal Code: 90040060;

[d] Hospital São Lucas, Pontifícia Universidade Católica do Rio Grande do Sul, Porto Alegre, Rio Grande do Sul, Brazil; Postal Code: 90610000;

[e] Secretaria Estadual de Saúde do Estado do Rio Grande do Sul, Porto Alegre, Rio Grande do Sul, Brazil; Postal Code: 90110150;

[f] Department of Psychiatry, Universidade Federal de Ciências da Saúde de Porto Alegre (UFCSPA), Porto Alegre, Rio Grande do Sul, Brazil; Postal Code: 90050170;

[g] Department of Internal Medicine, Universidade Federal do Rio Grande do Sul (UFRGS), Porto Alegre, Rio Grande do Sul, Brazil; Postal Code: 90035903;

[h] Cognitive and Behavioral Neurology Center, Division of Neurology, Hospital de Clínicas de Porto Alegre, Porto Alegre, Rio Grande do Sul, Brazil; Postal Code: 90035903.

Corresponding author: Raphael Machado Castilhos, Cognitive and Behavioral Neurology Center, Division of Neurology, Hospital de Clínicas de Porto Alegre, Porto Alegre, Rio Grande do Sul, Brazil. Ramiro Barcelos Street 2350, Portal Code: 90035903; Phone: 555133598182. ORCID: 0000-0002-1905-2084.

Supplementary Table 1. Description of medication for Alzheimer's disease prescribed in the state of Rio Grande do Sul.

Medication	n (%)<br>total: 2,382
**Donepezil (total)**	1,014 (42.6)
Donepezil 10 mg	798 (33.5)
Donepezil 5 mg	216 (9.1)
**Galantamine (total)**	429 (18)
Galantamine 24 mg	200 (8.4)
Galantamine 16 mg	137 (5.8)
Galantamine 8 mg	92 (3.9)
**Memantine (total)**	712 (29.9)
Memantine (monotherapy)	248 (10.4)
Memantine (association)	464 (19.5)
Memantine + donepezil 10 mg	215 (46.3)
Memantine + donepezil 5 mg	39 (8.4)
Memantine + galantamine 24 mg	54 (11.6)
Memantine + galantamine 16 mg	20 (4.3)
Memantine + galantamine 8 mg	14 (3)
Memantine + rivastigmine 6 mg	8 (1.7)
Memantine + rivastigmine 4.5 mg	7 (1.5)
Memantine + rivastigmine 3 mg	9 (1.9)
Memantine + rivastigmine 1.5 mg	12 (2.6)
Memantine + Rivastigmine 18 mg (patch)	73 (15.7)

Memantine + Rivastigmine 9 mg (patch)	13 (2.8)
**Rivastigmine (total)**	691 (29)
Rivastigmine 6 mg	54 (2.3)
Rivastigmine 4.5 mg	29 (1.2)
Rivastigmine 3 mg	41 (1.7)
Rivastigmine 1.5 mg	61 (2.6)
Rivastigmine 18 mg (patch)	415 (17.4)
Rivastigmine 9 mg (patch)	90 (3.8)
Rivastigmine 2 mg/mL (oral solution)	1 (0.04)
